# Crucial trials in neurosurgery: a must-know for every neurosurgeon

**DOI:** 10.1007/s10143-024-02358-4

**Published:** 2024-03-21

**Authors:** Oday Atallah, Khadeja Alrefaie, Yazeed Al Krinawe

**Affiliations:** 1 Department of Neurosurgery, Hannover Medical School, Carl-Neuberg Street. Nr. 1, 30625 Hannover, Germany; 2 Faculty of Medicine, Royal College of Surgeons in Ireland, Busaiteen, Bahrain

## Background

In the fast-progressing domain of neurosurgery, keeping up-to-date with pioneering clinical trials is not only advantageous, but also essential for delivering exceptional patient care. Young neurosurgeons and residents embarking on their careers must possess a profound comprehension of these crucial trials. These trials provide the foundation of evidence-based medicine, guiding clinical decision-making and influencing the future of patient treatments.

The objective of this study is to examine and assess the most impactful clinical trials in the field of neurosurgery. Through a detailed analysis and synthesis of the results from these trials, our aim is to create a helpful guide for young neurosurgeons, enabling them to remain updated and make well-informed decisions for their patients.

### Cerebral aneurysms

#### ISAT

The International Subarachnoid Aneurysm Trial (ISAT) of 2002 significantly influenced the management of ruptured intracranial aneurysms [1]. The trial compared two treatment methods: neurosurgical clipping and endovascular coiling. A total of 2,143 patients with ruptured intracranial aneurysms were enrolled and randomly assigned to either of the two treatments. The primary outcome was the proportion of patients who were dependent or dead (indicated by a modified Rankin scale score of 3–6) at one year post-treatment. Results showed a substantial benefit in favor of endovascular coiling, with 23.7% of patients in the coiling group versus 30.6% in the clipping group falling into the dependent or dead category [1]. This finding marked a pivotal moment in the approach to treating ruptured intracranial aneurysms, suggesting a significant advantage of endovascular coiling over neurosurgical clipping in improving patient independence and survival rates at the one-year mark.

#### ISUIA

The International Study of Unruptured Intracranial Aneurysms (ISUIA) Trial of 2003 provided critical insights into the management of unruptured intracranial aneurysms [2]. This extensive study, involving 4,060 patients across various centers, focused on comparing the natural history risks of unruptured aneurysms with the risks associated with surgical or endovascular interventions. Key findings included detailed data on rupture rates based on aneurysm size and location, revealing that small aneurysms in the anterior circulation had a very low risk of rupture, whereas larger aneurysms, particularly those in the posterior circulation, posed a higher risk. For instance, aneurysms less than 7 mm in size located in the anterior circulation had a very low risk of rupture. The study also emphasized the importance of individualized treatment decisions, influenced by factors such as patient age, aneurysm size, and location [2]. These findings have significantly influenced current practices, advocating for a nuanced and patient-specific approach in the management of unruptured intracranial aneurysms.

#### CARAT

The Cerebral Aneurysm Rerupture After Treatment (CARAT) study of 2008 aimed to identify predictors of rerupture following the treatment of ruptured intracranial aneurysms [3]. This study included 1,001 patients and used both coil embolization and surgical clipping as treatment methods. Key findings indicated that the degree of aneurysm occlusion post-treatment was a strong predictor of subsequent rupture. Specifically, the study found a graduated risk of rerupture based on the degree of occlusion: complete occlusion presented the lowest risk, while less than 70% occlusion showed the highest risk. While complete aneurysm occlusion of 100% post initial treatment did not guarantee rerupture nonoccurrence, however, the risk of rerupture was only 1.1% in their study group with all events occurring during the first year [3]. This study underlined the importance of achieving as complete an occlusion as possible during initial treatment to reduce the risk of rerupture.

#### SUAVe

The Small Unruptured Intracranial Aneurysm Verification study (SUAVe) of 2010 focused on the natural history and optimal management of incidentally discovered small unruptured intracranial aneurysms less than 5 mm in diameter [4]. The study included 540 aneurysms in 446 patients, with a follow-up period averaging 41 months. Key findings revealed the average annual risks of rupture for small unruptured aneurysms to be relatively low at 0.54% overall, with 0.34% for single aneurysms and 0.95% for multiple aneurysms. Significant predictive factors for rupture included patient age below 50 years, aneurysm diameter of 4.0 mm or larger, hypertension, and aneurysm multiplicity [4]. This study underscores the importance of individualized treatment decisions for small unruptured aneurysms, particularly in younger patients with hypertension and multiple aneurysms of larger size.

#### BRAT

The Barrow Ruptured Aneurysm Trial (BRAT) of 2012 was a significant study in the field of neurosurgery that focused on comparing the efficacy and safety of microsurgical clipping and endovascular coil embolization in treating acutely ruptured cerebral aneurysms [5]. Enrolling 470 patients, the trial aimed to determine if one treatment method was superior to the other by analyzing clinical and angiographic outcomes. The study’s primary outcome was based on patient results at 1 year post-treatment, assessed using the modified Rankin Scale (mRS). A key finding was that patients assigned to coil embolization had fewer poor outcomes compared to those assigned to surgical clipping [5]. This result supported the growing inclination towards endovascular treatment, emphasizing the need for quality surgical clipping as an alternative treatment modality.

### Intracerebral hemorrhage

#### STICH I

The Surgical Trial in Intracerebral Haemorrhage (STICH) I Trial (2005) was a landmark study in the field of neurosurgery focusing on spontaneous supratentorial intracerebral hemorrhages [6]. This randomized trial compared early surgery (haematoma evacuation within 24 h of randomization) with initial conservative treatment. The study involved 1,033 patients from 83 centers across 27 countries. The primary outcome measured was the six-month prognosis using the Glasgow outcome scale. The results showed no significant overall benefit from early surgery compared to initial conservative treatment [6]. The trial’s findings have been influential in guiding treatment approaches for spontaneous supratentorial intracerebral hemorrhages, highlighting the complexity and need for individualized patient assessment in these cases.

#### STICH II

The STICH II Trial (2013) followed up on the STICH I Trial to further investigate the efficacy of early surgery for spontaneous supratentorial lobar intracerebral hemorrhages [7]. This randomized trial involved 601 patients, comparing early surgical hematoma evacuation within 12 h of randomization plus medical treatment against initial conservative treatment. The primary outcome was based on the Extended Glasgow Outcome Scale (GOSE) at 6 months. The findings showed that early surgery did not significantly increase the rate of death or disability at 6 months compared to conservative treatment, suggesting a small potential survival advantage for patients with superficial intracerebral hemorrhage without intraventricular hemorrhage [7].

#### INTERACT-2

The Intensive Blood Pressure Reduction in Acute Cerebral Hemorrhage Trial (INTERACT-2 Trial) (2015) was a significant study in the field of neurosurgery focusing on acute intracerebral hemorrhage (ICH) [8]. The trial investigated the effects of intensive blood pressure (BP) lowering in patients with acute ICH. The study involved 2,839 patients who were randomized to receive either intensive BP lowering treatment (target systolic BP of 140 mm Hg) or guideline-recommended BP lowering treatment. The primary outcome was physical function across all seven levels of the modified Rankin Scale at 90 days. The trial’s results suggested that intensive BP lowering is beneficial across a wide range of baseline systolic BP levels, and a target systolic BP level of 130–139 mm Hg is likely to provide maximum benefit in acute ICH [8].

#### ATACH-2

The Antihypertensive Treatment of Acute Cerebral Hemorrhage II (ATACH-2) Trial (2016) conducted a comprehensive investigation into the effectiveness of intensive blood pressure lowering in patients with acute intracerebral hemorrhage [9]. The study involved 1,000 participants, who were randomized into two groups: one receiving intensive treatment to achieve a target systolic blood pressure of 110 to 139 mm Hg, and the other receiving standard treatment with a target of 140 to 179 mm Hg. A primary focus of the trial was to assess the rate of death or disability at 3 months post-treatment. The findings indicated that 38.7% of participants in the intensive-treatment group and 37.7% in the standard-treatment group experienced death or disability. This suggested no significant difference in outcomes between the two groups. Additionally, the trial found that rapid lowering of blood pressure in patients with acute intracerebral hemorrhage did not result in a lower rate of death or disability compared to standard reduction targets [9]. These results have important implications for the management of blood pressure in the acute phase of intracerebral hemorrhage, indicating that more aggressive blood pressure reduction may not confer additional benefits in terms of reducing death or disability.

#### CLEAR III

The Clot Lysis: Evaluating Accelerated Resolution of Intraventricular Hemorrhage (CLEAR) III Trial (2017) was an influential study in the field of neurosurgery, specifically focusing on intraventricular hemorrhage (IVH) treatment [10]. Conducted between September 2009 and January 2015, this randomized trial involved 500 patients, dividing them into two groups: 249 patients received alteplase, and 251 patients received saline. The primary objective was to assess functional outcomes, measured by the modified Rankin Scale (mRS), at 180 days post-treatment. One of the key findings is the primary efficacy outcome (good functional outcome, defined as mRS ≤ 3) that was similar in both the alteplase group (48%) and the saline group (45%), indicating no significant difference in the primary outcome measure. After adjustments for intraventricular hemorrhage size and thalamic intracerebral hemorrhage, a slight difference of 3.5% in favor of alteplase was noted, but this was not statistically significant. Notably, the alteplase group exhibited a lower case fatality rate (18% vs. 29% in the saline group) at 180 days, suggesting a potential survival benefit of alteplase treatment. However, a higher proportion of patients in the alteplase group had severe disability (mRS 5, indicating severe disability requiring constant nursing care and attention). The study also observed lower rates of ventriculitis and serious adverse events in the alteplase group compared to the saline group, suggesting a safety advantage for alteplase. The rate of symptomatic bleeding was similar in both groups. These results suggest that while alteplase did not significantly improve functional outcomes in patients with intraventricular hemorrhage compared to saline, it was associated with a lower case fatality rate and fewer serious adverse events. However, a higher proportion of survivors in the alteplase group had severe disability [10]. The trial’s findings highlight the complexities in managing intraventricular hemorrhage and point to the need for further research to optimize treatment strategies.

#### MISTIE III

The Minimally Invasive Surgery with Thrombolysis in Intracerebral Haemorrhage Evacuation (MISTIE) III Trial, conducted in 2019, was a pivotal study in neurosurgery focused on evaluating the efficacy of a minimally invasive surgery plus alteplase in treating intracerebral hemorrhage [11]. This trial involved 506 patients, divided into two groups: one underwent the MISTIE procedure, and the other received standard medical care. The primary aim was to assess functional outcomes using the modified Rankin Scale (mRS) at 365 days. The findings indicated that there was no significant difference in functional outcomes between the two groups. However, the trial noted a slightly lower mortality rate in the MISTIE group compared to standard care, suggesting a potential survival benefit. Additionally, the study found that the MISTIE procedure was safe and did not increase the risk of serious bleeding or infection. Despite these findings, the trial concluded that the MISTIE procedure, as performed in this study, could not be recommended as a standard treatment to improve functional outcomes in all patients with intracerebral hemorrhage, highlighting the need for further research and refinement of the technique [11].

### Middle cerebral artery infarction

#### DECIMAL

The Decompressive Craniectomy In Malignant MCA Infarction (DECIMAL) Trial of 2007 was a significant study in the field of neurosurgery focusing on malignant middle cerebral artery (MCA) infarction [12]. The trial aimed to assess the efficacy of early decompressive craniectomy in patients with malignant MCA infarction. It involved 38 patients between 18 and 55 years of age, randomized to either standard medical therapy or medical therapy plus decompressive craniectomy. The primary outcome measured was the development of moderate disability (modified Rankin scale score ≤ 3) at 6 months’ follow-up. The results indicated a notable reduction in mortality and an increase in the number of patients with moderate disability in the surgery group compared to the no-surgery group [12]. The trial provided significant insights into the benefits of early decompressive craniectomy in young patients with malignant MCA infarction, emphasizing its potential in improving survival and functional outcomes.

#### DESTINY

The Decompressive Surgery for the Treatment of Malignant Infarction of the Middle Cerebral Artery (DESTINY) Trial of 2007 focused on the treatment of malignant middle cerebral artery infarction through decompressive surgery (hemicraniectomy) [13]. This randomized controlled trial involved 32 patients and aimed to assess the impact of hemicraniectomy on 30-day mortality and 6- and 12-month functional outcomes. The results demonstrated a significant reduction in mortality for the surgical group compared to conservative therapy, with 88% of patients in the surgical arm surviving at 30 days compared to 47% in the conservative treatment group. However, the primary endpoint, which was functional outcome at 6 months measured by the modified Rankin Scale score dichotomized to 0 to 3 versus 4 to 6, did not show statistical superiority of hemicraniectomy [13]. Despite this, the trial provided valuable insights into the potential benefits of decompressive surgery in reducing mortality in cases of large hemispheric stroke.

#### HAMLET

The HAMLET Trial (Hemicraniectomy After Middle Cerebral Artery infarction with Life-threatening Edema Trial) of 2009 was a multicenter, randomized trial focusing on the effects of surgical decompression in patients with space-occupying hemispheric infarctions [14]. The trial included 64 patients who were randomly assigned to either surgical decompression or best medical treatment within 4 days of stroke onset. The primary outcome was functional outcome at 1 year, measured by the modified Rankin scale. The results showed that surgical decompression did not significantly affect the primary outcome measure, but it did reduce case fatality. The trial provided insights into the timing and effectiveness of surgical intervention in patients with space-occupying hemispheric infarction, highlighting the importance of early intervention within 48 h of stroke onset for improving outcomes [14].

#### DESTINY II

The DESTINY II Trial of 2014 extended the investigation of decompressive surgery in patients with malignant middle cerebral artery (MCA) infarction [15]. This trial focused on older patients (ages 61 and above) and compared early hemicraniectomy to conservative treatment. It involved 112 patients, with findings showing that hemicraniectomy significantly increased survival without severe disability. The survival rate without severe disability was 38% in the hemicraniectomy group compared to 18% in the control group. However, most survivors required assistance with most bodily needs, with a significant portion experiencing severe disability [15]. The trial’s results highlight the complex decision-making required in treating older patients with malignant MCA infarction, balancing survival against potential disability.

### Traumatic brain injury

#### CRASH 1

The CRASH-1 Trial of 2004 focused on the effect of intravenous corticosteroids in adults with clinically significant head injury [16]. The trial involved 10,008 patients who were randomly allocated to receive either a 48-h infusion of corticosteroids (methylprednisolone) or a placebo. The primary outcomes were death within 2 weeks of injury and death or disability at 6 months. The results showed that compared with placebo, the risk of death within 2 weeks was higher in the group allocated corticosteroids. This significant finding led to the conclusion that corticosteroids do not reduce mortality and in fact, might increase the risk of death within the first two weeks post-injury [16].

#### CRASH 2

The CRASH-2 Trial (2010) was a significant study in the field of trauma care, specifically focused on the effects of tranexamic acid in trauma patients with significant hemorrhage [17]. This large, randomized, placebo-controlled trial included over 20,000 adult trauma patients from 274 hospitals in 40 countries. The study assessed whether early administration of tranexamic acid (within 8 h of injury) would reduce deaths, vascular occlusive events, and the need for blood transfusion. The results showed that tranexamic acid significantly reduced all-cause mortality and the risk of death due to bleeding. Importantly, the trial found that tranexamic acid safely reduced the risk of death in bleeding trauma patients without increasing the risk of vascular occlusive events. The study’s findings have had substantial implications for the management of trauma patients with significant bleeding [17].

#### DECRA

The DECRA Trial (Decompressive Craniectomy in Diffuse Traumatic Brain Injury) of 2011 was a pivotal study in the field of neurosurgery, focusing on the efficacy of decompressive craniectomy in patients with severe traumatic brain injury and refractory raised intracranial pressure [18]. The study randomized 155 adults to undergo either bifrontotemporoparietal decompressive craniectomy or receive standard care. The results showed that while patients undergoing craniectomy had less time with intracranial pressures above the treatment threshold and fewer days in the ICU, they also had worse outcomes on the Extended Glasgow Outcome Scale and a greater risk of an unfavorable outcome. This trial highlighted the complexities in managing severe traumatic brain injury and indicated that early decompressive craniectomy, although reducing intracranial pressure, might not improve functional outcomes [18].

#### CRASH 3

The CRASH-3 Trial, conducted in 2019, focused on the use of tranexamic acid in patients with traumatic brain injury (TBI) [19]. This large-scale, randomized, placebo-controlled trial involved 12,737 patients across 29 countries. The primary aim was to evaluate the effect of tranexamic acid on head injury-related death in hospital within 28 days of injury for patients treated within 3 h of injury. The trial showed that treatment with tranexamic acid within 3 h of injury reduced the risk of head injury-related death, particularly in patients with mild-to-moderate head injury, but not in those with severe head injury. Additionally, the risk of vascular occlusive events and seizures was similar between the tranexamic acid and placebo groups, indicating the safety of this treatment in TBI patients. The CRASH-3 trial’s results suggest that early administration of tranexamic acid can be a beneficial intervention in TBI cases, especially for those with less severe injuries [19].

### Arteriovenous malformations

#### ARUBA

The ARUBA Trial (A Randomized trial of Unruptured Brain Arteriovenous malformations) in 2014 was a groundbreaking study in neurovascular research [20]. It aimed to compare the outcomes of medical management alone versus medical management with interventional therapy for unruptured brain arteriovenous malformations. The trial enrolled 226 patients across 39 clinical sites in nine countries. The primary outcome was a composite of death or symptomatic stroke. One of the key findings from the ARUBA Trial is that the risk of death or stroke was significantly lower in the medical management group than in the interventional therapy group. This result was observed over a mean follow-up period of 33.3 months. The trial showed a higher number of strokes and neurological deficits unrelated to stroke in patients allocated to interventional therapy compared to medical management. The medical management group had a lower risk of death and neurological disability (modified Rankin scale ≥ 2) compared to the interventional group. The trial emphasized the superiority of medical management alone over medical management with interventional therapy in preventing death or stroke in patients with unruptured brain arteriovenous malformations. The ARUBA Trial’s results have significant implications for the management of unruptured brain arteriovenous malformations, indicating that conservative medical management may be a safer approach than interventional treatments in certain cases [20].

### Myelomeningocele

#### MOMS

The Management of Myelomeningocele Study (MOMS), conducted in 2011, was a pivotal trial comparing prenatal versus postnatal repair of myelomeningocele, a common form of spina bifida [21]. This study involved 183 patients, with outcomes evaluated in 158 patients at 12 months and 134 patients at 30 months. Key findings include that the prenatal surgery group showed a significant reduction in the need for cerebrospinal fluid shunt placement and improvements in several secondary outcomes, including hindbrain herniation, at 12 months. At 30 months, the prenatal surgery group demonstrated better motor outcomes and composite scores of mental development and motor function. Despite these benefits, prenatal surgery was associated with increased risks of preterm delivery and uterine dehiscence at delivery [21]. These results indicate that prenatal surgery for myelomeningocele can reduce the need for shunting and improve motor outcomes but comes with maternal and fetal risks.

### Spinal neurosurgery

#### SPORT 2006

The Spine Patient Outcomes Research Trial (SPORT) of 2006 was a pivotal study in spinal neurosurgery, focusing on the treatment of lumbar disk herniation [22]. Conducted across 13 medical centers in the United States, the trial included 743 patients, who chose between standard open diskectomy (surgery) and usual nonoperative care. The trial’s goal was to assess the effectiveness of these treatments in improving symptoms of radiculopathy secondary to lumbar disk herniation. Patients in both treatment groups experienced substantial improvement over time, but those who underwent surgery reported significantly greater improvements. The benefits of surgery were noticeable as early as 6 weeks and were sustained for at least two years. This suggests that surgery offers a more rapid and possibly more effective treatment for symptoms of lumbar disk herniation, especially in terms of pain relief and physical function. Importantly, the study’s findings emphasize the clinical significance of changes observed in quality-of-life measures. The SPORT results, particularly from the observational cohort, exceeded the thresholds for clinically important differences in scales like the SF-36 subscales and the Oswestry Disability Index (ODI), reinforcing the relevance and impact of the surgical intervention in real-world settings. Overall, the SPORT Trial provides crucial insights into the management of lumbar disk herniation, underlining the effectiveness of surgical intervention, especially in cases where rapid symptom relief and improvement in quality of life are priorities. The study also highlights the importance of patient choice and individualized treatment approaches in managing lumbar disk herniation [22].

#### SPORT 2007

The SPORT Trial of 2007 further advanced our understanding of the treatment for lumbar degenerative spondylolisthesis, focusing on surgical versus non-surgical approaches [23]. The study, spanning across 13 centers in the United States, enrolled 607 patients. Patients were offered the choice of standard decompressive laminectomy (with or without fusion) or usual non-surgical care. The primary outcome measures were the SF-36 bodily pain and physical function scores and the modified Oswestry Disability Index, assessed over a two-year period. Key findings includes the as-treated analysis across both cohorts showed significant advantages for surgery at 3 months, with this benefit persisting at 1 year and only slightly diminishing at 2 years. Treatment effects at 2 years were significant for bodily pain, physical function, and the Oswestry Disability Index. The study found minimal evidence of harm from either treatment. This trial highlighted the substantial improvement in pain and function over two years in patients treated surgically compared to those receiving non-surgical care, highlighting the importance of individualized treatment decisions in managing lumbar degenerative spondylolisthesis [23].

#### SPORT 2008

The 2008 SPORT Trial, an extension of the 2006 and 2007 studies, further examined treatments for lumbar disc herniation with a focus on long-term outcomes over a four-year period [24]. Conducted at 13 medical centers across the U.S., the trial included a total of 634 patients, with 278 in the randomized group and 356 in the observational group. The study continued to compare surgical intervention (standard open diskectomy) with nonoperative care. The results reinforced earlier findings that surgery provided more substantial improvements in pain and function compared to nonoperative care. However, both groups showed significant improvements over time. The extended follow-up of this trial provided valuable insights into the long-term benefits and potential limitations of each treatment option, emphasizing the importance of considering patient choice and long-term implications in the management of lumbar disc herniation [24].

#### SLIP

The Spinal Laminectomy versus Instrumented Pedicle Screw (SLIP) Trial of 2016, a significant study in spinal neurosurgery, focused on comparing the outcomes of lumbar laminectomy with and without fusion in patients with lumbar spondylolisthesis [25]. The trial involved 130 patients who were screened for eligibility, with 106 patients eventually participating. Among these, 66 consented to randomization, and 40 chose their preferred surgical method but agreed to be part of an observation group. This study specifically aimed to determine if the addition of instrumented fusion to laminectomy would result in greater improvement in patients’ physical health-related quality of life, as measured by the SF-36 physical-component summary score, over a period of two years. Additionally, the study evaluated outcomes at 3 and 4 years. The key findings of the SLIP Trial were that lumbar laminectomy plus fusion was associated with a slightly greater, but clinically meaningful, improvement in physical health-related quality of life compared to laminectomy alone, sustained over 2, 3, and 4 years after surgery. While the between-group differences in the SF-36 physical-component summary score were small, they were considered clinically meaningful. There were no significant differences between the groups in terms of reductions in the Oswestry Disability Index score, a secondary measure of disability related to back pain. Patients undergoing fusion experienced more blood loss and longer hospital stays compared to those in the decompression-alone group. The cumulative rate of reoperation was lower in the fusion group (14%) compared to the decompression-alone group (34%) [25]. These results highlight the nuanced decision-making process in treating lumbar spondylolisthesis, particularly when weighing the benefits of additional fusion against potential risks and postoperative outcomes.

#### NECK

The NEtherlands Cervical Kinetics (NECK) Trial of 2019 was a significant study in spinal neurosurgery focusing on the effectiveness of different surgical techniques for cervical disc herniation [26]. This double-blinded randomized controlled trial compared anterior cervical discectomy with or without interbody fusion and arthroplasty. It involved 109 patients with one-level herniated disc, randomized to receive one of three treatments: anterior cervical discectomy with disc prosthesis (ACDA), anterior cervical discectomy with fusion (ACDF) using an intervertebral cage, or anterior cervical discectomy without fusion (ACD). Clinical and radiological outcomes were measured up to two years after surgery, including Neck Disability Index (NDI), Visual Analogue Scale (VAS) for neck and arm pain, and radiographic evaluation of cervical curvature and adjacent segment degeneration. The results indicated no significant differences in clinical outcomes among the three surgical methods. All treatment groups showed improvement in NDI and VAS scores for arm and neck pain, with no clear advantage of one technique over the others [26]. This suggests that single-level ACD without an intervertebral device may be a reasonable alternative to ACDF or ACDA, challenging the perceived necessity of intervertebral devices in certain cases of cervical disc herniation.

## Discussion

**Fig. 1:**
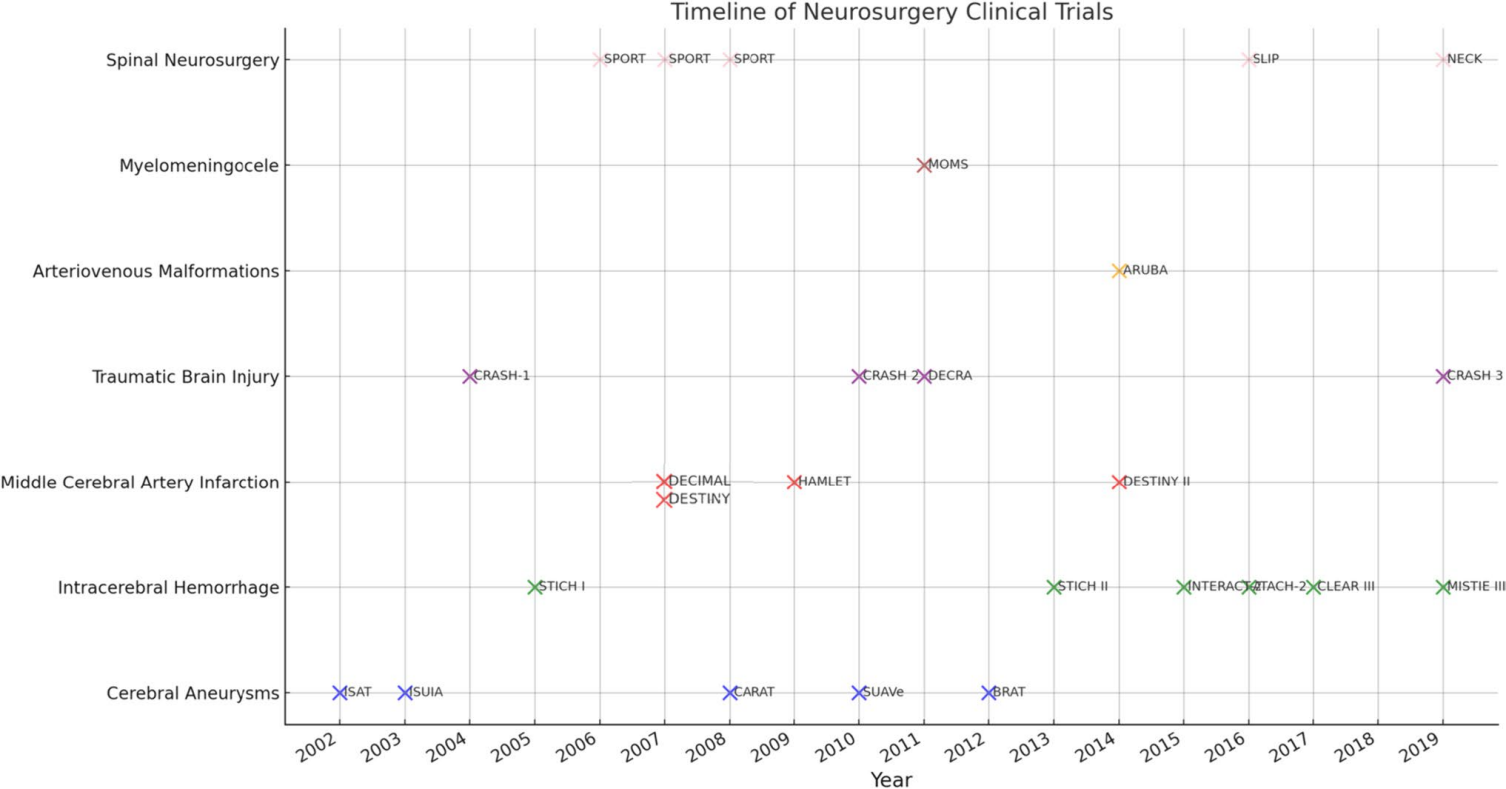
Timeline of pivotal clinical trials in the field of neurosurgery

The landscape of neurosurgery is continually evolving, with clinical trials playing a pivotal role in shaping the future of patient care. For neurosurgeons, especially those in the early stages of their careers, an in-depth understanding of these trials is not just beneficial but essential. These trials provide a foundation for evidence-based practice, guiding neurosurgeons in making informed decisions that significantly impact patient outcomes. For young neurosurgeons and residents, this knowledge is critical in making informed clinical decisions. By being aware of the latest findings, they can choose treatment options that are supported by robust clinical evidence, thereby enhancing the quality of patient care (Table 1, Fig. 1).

**Table 1:** Oulining clinical trials, the specific intervention techniques used, primary and secondary outcomes and key findings

Study	Year	Focus	Participants	Intervention	Primary outcomes	Secondary outcomes	Key findings
Clinical trials of cerebral aneurysms							
ISAT	2002	Ruptured Intracranial Aneurysms	2,143	Neurosurgical Clipping vs. Endovascular Coiling	Dependency or death at 1 year (mRS score 3–6)	-	Lower dependency or death rate in the endovascular coiling group; significant advantage of coiling over clipping for patient independence and survival at 1 year
ISUIA	2003	Unruptured Intracranial Aneurysms	4,060	Surgical or Endovascular Treatment vs. Conservative Management	Rupture rates based on aneurysm size/location	-	Rupture rates based on aneurysm size and location; comparison of natural history risks with intervention risks
CARAT	2008	Rerupture Post-Treatment of Ruptured Aneurysms	1,001	Coil Embolization and Surgical Clipping	Predictors of rerupture after treatment	-	Degree of aneurysm occlusion post-treatment as a strong predictor of rerupture; complete occlusion presented the lowest risk
SUAVe	2010	Small Unruptured Intracranial Aneurysms	540 aneurysms in 446 patients	Observation vs. Intervention	Average annual risks of rupture for small unruptured aneurysms	-	Low annual rupture rates (0.54% overall); significant factors for rupture include younger age, larger aneurysm size, hypertension, and aneurysm multiplicity​​
BRAT	2012	Acutely Ruptured Cerebral Aneurysms	470	Microsurgical Clipping vs. Endovascular Coil Embolization	Patient outcomes at 1 year (mRS)	-	Fewer poor outcomes (mRS score > 2) in coil group (20.4%) compared to clip group (33.9%) at 1 year​​
Clinical trials of intracerebral hemorrhage							
STICH I	2005	Supratentorial Intracerebral Hemorrhage	1,033	Early Surgery vs. Conservative Treatment	Six-month prognosis (Glasgow outcome scale)	-	No significant overall benefit from early surgery compared to conservative treatment
STICH II	2013	Supratentorial Lobar Intracerebral Hemorrhage	601	Early Surgery vs. Conservative Treatment	Death or disability at 6 months (GOSE)	-	No significant increase in death or disability with early surgery; small potential survival advantage
INTERACT-2	2015	Acute Intracerebral Hemorrhage	2,839	Intensive BP Lowering vs. Guideline-Recommended Treatment	Physical function (modified Rankin Scale) at 90 days	-	Intensive BP lowering beneficial, target systolic BP 130–139 mm Hg likely most beneficial
ATACH-2	2016	Acute Cerebral Hemorrhage	1,000	Intensive vs. Standard BP Lowering	Death or disability at 3 months	-	No significant difference in death or disability between intensive and standard BP lowering
CLEAR III	2017	Intraventricular Hemorrhage	500	Alteplase vs. Saline Irrigation	Functional outcomes at 180 days (mRS)	Lower case fatality in alteplase group	No significant improvement in functional outcomes with alteplase; lower case fatality rate in alteplase group
MISTIE III	2019	Intracerebral Hemorrhage	506	Minimally Invasive Surgery plus Alteplase vs. Standard Medical Care	Functional outcomes (mRS) at 365 days	Lower mortality rate in MISTIE group	No significant difference in functional outcomes; slightly lower mortality in MISTIE group
Clinical trials of middle cerebral artery infarctions							
DECIMAL	2007	Malignant MCA Infarction	38	Decompressive Craniectomy vs. Medical Therapy	Moderate disability development (mRS ≤ 3) at 6 months	-	Reduction in mortality and increased survival without severe disability in surgery group
DESTINY	2007	Malignant MCA Infarction	32	Hemicraniectomy vs. Conservative Therapy	30-day mortality and 6–12 month functional outcomes	-	Significant reduction in mortality for surgical group, but no statistical superiority in functional outcome
DESTINY II	2014	Malignant MCA Infarction in Older Patients	112	Early Hemicraniectomy vs. Conservative Treatment	Survival without severe disability	-	Increased survival without severe disability in hemicraniectomy group, but most survivors required assistance
HAMLET	2009	Space-occupying Hemispheric Infarctions	64	Surgical Decompression vs. Medical Treatment	Functional outcome at 1 year (modified Rankin scale)	Reduced case fatality	No effect on primary outcome measure but reduced case fatality. Early intervention within 48 h improves outcomes​​
Clinical trials of traumatic brain injury							
CRASH-1	2004	Head Injury	10,008	Corticosteroids vs. Placebo	Death within 2 weeks and death/disability at 6 months	-	Increased risk of death with corticosteroids, no reduction in mortality within 2 weeks of head injury​​
CRASH 2	2010	Bleeding Trauma Patients	20,211	Tranexamic Acid vs. Placebo	Deaths, vascular occlusive events, blood transfusion need	-	Significant reduction in all-cause mortality and death due to bleeding with tranexamic acid​​
DECRA	2011	Traumatic Brain Injury	155	Craniectomy vs. Standard Care	Outcomes on Extended Glasgow Outcome Scale, risk of unfavorable outcome	-	Less time with increased intracranial pressures in craniectomy group, but worse functional outcomes compared to standard care​​
CRASH 3	2019	Traumatic Brain Injury	12,737	Tranexamic Acid vs. Placebo	Head injury-related death in hospital within 28 days	-	Reduced risk of head injury-related death with tranexamic acid in patients with mild-to-moderate head injury, no clear evidence of reduction in severe head injury​​
Clinical trial of arteriovenous malformations							
ARUBA	2014	Unruptured Brain AVMs	226	Medical Management vs. Interventional Therapy	Death or symptomatic stroke	-	Medical management alone was superior to medical management with interventional therapy in preventing death or stroke. Lower risk of death/stroke and neurological deficits with medical management. Higher number of strokes and neurological deficits unrelated to stroke in interventional therapy group
Clinical trials of myelomeningocele							
MOMS Trial	2011	Myelomeningocele Repair	183	Prenatal vs. Postnatal Surgery	Need for cerebrospinal fluid shunt placement and improvements in secondary outcomes	Increased risks of preterm delivery and uterine dehiscence	Prenatal surgery reduced the need for shunting and improved motor outcomes at 30 months but increased risks of preterm delivery and uterine dehiscence
Clinical trials of spinal neurosurgery							
SPORT	2006	Lumbar Disk Herniation	743	Surgery vs. Nonoperative Care	Improvements in bodily pain, physical function, Oswestry Disability Index	-	Surgery reported greater improvements in pain and function than nonoperative care
SPORT	2007	Lumbar Disk Herniation	607	Surgery vs. Nonoperative Care	SF-36 bodily pain and physical function scores, Oswestry Disability Index over two years	-	Surgically treated patients showed substantially greater improvement in pain and function over two years compared to nonsurgical treatment
SPORT	2008	Lumbar Disk Herniation	634	Surgery vs. Nonoperative Care	Long-term benefits and limitations of surgery vs. nonoperative care	-	Surgery provided more significant long-term improvements in pain and function
SLIP	2016	Lumbar Spinal Stenosis	106	Decompression Alone vs. Decompression with Fusion	Improvement in physical health-related quality of life (SF-36) over two years	Cumulative rate of reoperation	No significant differences in outcomes; decompression alone may be a viable option
NECK	2019	Cervical Radiculopathy	112	ACD vs. ACDF vs. ACDA	Clinical and radiological outcomes up to two years	Neck Disability Index, Visual Analogue Scale for neck and arm pain	No significant differences in clinical outcomes among the three surgical interventions

Neurosurgeons, through familiarization with these key trials, can integrate the latest evidence into their clinical practice. This approach not only improves patient outcomes but also ensures that treatment decisions are up-to-date and in line with the current standards of care in the neurosurgical community. Trials like SUAVe and STICH have underscored the importance of individual patient factors in treatment planning (4, 6, 7). Understanding these trials helps neurosurgeons appreciate the nuances of personalized patient care, recognizing that each patient may require a different approach based on their specific medical conditions and overall health status (Table 2).

**Table 2:** Scale Utilization; the metrics for assessing outcomes in neurosurgical trials

Scale	Purpose	Used in trials
Modified Rankin Scale (mRS)	Assesses the degree of disability or dependence in daily activities	ISAT, BRAT, MISTIE III, CLEAR III, DECIMAL, DESTINY, HAMLET, STICH I, STICH II
Glasgow Outcome Scale	Measures the overall outcome, including death, vegetative state, and levels of disability	STICH I, DECRA
Oswestry Disability Index (ODI)	Evaluates the degree of disability related to back pain	SPORT Trials
SF-36 (Physical and Mental Component Summary)	Assesses health-related quality of life	SPORT Trials
Visual Analogue Scale (VAS) for Pain	Measures pain intensity	NECK
Extended Glasgow Outcome Scale (GOSE)	Provides a more detailed assessment of outcomes, especially in cases of brain injury	STICH II, DECRA

As neurosurgery becomes increasingly intertwined with technological advancements, young neurosurgeons need to stay informed about the latest techniques and tools. Trials that evaluate the efficacy of new technologies or surgical techniques, such as MISTIE III, provide invaluable insights into the benefits and limitations of these innovations (11). These trials often highlight the risks associated with various neurosurgical interventions. For neurosurgeons, understanding these risks is crucial in practicing ethically and in managing patient expectations. This knowledge is fundamental in obtaining informed consent and in discussing potential outcomes and complications with patients.

The diversity of trials, covering a range of neurosurgical subspecialties, prepares young neurosurgeons for the complexity they will encounter in their practice. This preparation is essential for developing the confidence and competence required to handle challenging cases. This awareness can inspire them to contribute to ongoing research efforts, whether through participating in future trials or through academic pursuits that seek to further the field. Familiarizing oneself with these trials is a part of lifelong learning and professional development. For young neurosurgeons and residents, this commitment to continuous education is pivotal in building a successful and fulfilling career in neurosurgery.

The knowledge of pivotal clinical trials in neurosurgery is indispensable for young neurosurgeons and residents. It aids them in making informed decisions, ensuring the best possible patient outcomes, and contributes to their professional growth and development. As the field of neurosurgery continues to evolve, staying abreast of these trials will be crucial for the next generation of neurosurgeons to practice effectively and ethically.

## Implications for clinical practice

The pivotal clinical trials we’ve explored hold substantial implications for neurosurgical practice, impacting treatment protocols, patient management strategies, and the incorporation of new surgical techniques and technologies [27]. These studies have been instrumental in shaping contemporary treatment protocols, as evidenced by the shift towards endovascular coiling in cerebral aneurysms management following the ISAT and BRAT trials [1, 5, 27, 28].

This shift is not just a change in technique but represents a broader move towards less invasive procedures in neurosurgery, emphasizing patient safety and recovery. For instance, a study by Gupta et al. in 2018 discusses the recent introduction of new devices after those trials, such as flow diverter stents, microstents, bifurcation devices, double-lumen balloon catheters, and microcoils, which have proved effective in overcoming the limitations of traditional aneurysm coiling [27]. Also, a study by Eboli et al. indicates a treatment paradigm shift in MCA aneurysm treatment from surgical treatment to endovascular treatment over the past decade [29]. Also, a study by Alawi et al. found that endovascular coiling was associated with fewer deaths and shorter hospital stays than clip placement, and the trend of hospitals’ use of coiling operations has increased in recent years [30]. The trials also highlight the importance of personalized patient management, with trials like SUAVe emphasizing the need for individualized approaches based on specific factors such as aneurysm size and patient age [4, 31].

Moreover, advancements in surgical techniques and technologies are vividly illustrated in these trials, with studies like MISTIE III highlighting the potential of minimally invasive surgery in intracerebral hemorrhage [11]. The emphasis on timely intervention, particularly in acute conditions as shown in the DECIMAL and DESTINY trials, has influenced urgent decision-making in neurosurgery [12, 13, 15]. For instance, after DECIMAL and DESTINY trials, Heiss W et al. published a study on the importance of early severe neurological symptoms in predicting the course of malignant MCA infarction and it emphasizes that early diagnosis with neuroimaging can predict the condition with high sensitivity, highlighting the need for timely intervention [32]. Additionally, a recent study in 2023 titled “It is all about timing: decompressive hemicraniectomy for malignant middle-cerebral-artery infarction” by Macha K et al. includes large analysis of randomized patients shows that decompressive surgery leads to higher rates of survival and functional outcomes in patients with malignant MCA infarction, highlighting the critical role of timing in surgical intervention and emphasizing the findings of those trials [33].

Furthermore, these trials contribute to a deeper understanding of the risk–benefit analysis of various neurosurgical interventions, aiding neurosurgeons in making more informed decisions and effectively communicating these with patients. The extended follow-up in studies like the 2008 SPORT Trial also provides valuable insights into long-term patient care and monitoring, shaping follow-up care strategies and patient counseling about long-term outcomes [24].

These trials collectively signify a dynamic and evolving landscape in neurosurgery, where traditional practices are continually being challenged and refined. They represent a commitment to advancing patient care through innovation, whether through the adoption of new surgical techniques, the integration of cutting-edge technology, or the exploration of novel therapeutic approaches. This ongoing evolution ensures that neurosurgical practice remains at the forefront of medical science, consistently improving outcomes and quality of life for patients.

## Conclusion

Familiarity with these pivotal studies hold particular significance for young neurosurgeons and residents. Such knowledge not only enhances clinical decision-making and patient outcomes but also fosters a deeper appreciation for evidence-based practice. For young professionals, understanding these trials is fundamental to navigating the complexities of neurosurgery, managing diverse patient needs, and integrating cutting-edge techniques into their practice. Moreover, this awareness prepares them for active participation in the ongoing evolution of neurosurgery, encouraging a commitment to lifelong learning and continuous professional development. As the field advances, the role of these trials in educating and guiding young neurosurgeons will be instrumental in ensuring the continued progress and innovation in neurosurgical care.

## Data Availability

No datasets were generated or analysed during the current study.
